# Comparative Study of Novel Methods for Olive Leaf Phenolic Compound Extraction Using NADES as Solvents

**DOI:** 10.3390/molecules28010353

**Published:** 2023-01-01

**Authors:** Paraskevi Siamandoura, Constantina Tzia

**Affiliations:** Laboratory of Food Chemistry and Technology, School of Chemical Engineering, National Technical University of Athens, 5 Iroon Polytechniou St., 15780 Zografou, Greece

**Keywords:** olive leaves, phenolic compounds, NADES, assisted extraction methods

## Abstract

Natural deep eutectic solvents (NADES) composed of choline chloride with maltose (CMA), glycerol (CGL), citric (CCA) and lactic acid (CLA) combined with microwave (MAE), ultrasound (UAE), homogenate (HAE) and high hydrostatic pressure (HHPAE)-assisted extraction methods were applied to recover and compare olive leaf phenolic compounds. The resultant extracts were evaluated for their total phenol content (TPC), phenolic profile and antioxidant activity and compared with those of water and ethanol:water 70% *v*/*v* extracts. HAE was proven to be the most efficient method for the recovery of olive leaf phenolic compounds. The highest TPC (55.12 ± 1.08 mg GAE/g d.w.) was found in CCA extracts after HAE at 60 °C and 12,000 rpm, and the maximum antioxidant activity (3.32 ± 0.39 g d.w./g DPPH) was found in CGL extracts after UAE at 60 °C for 30 min. The TPCs of ethanol extracts were found to be higher than those of NADES extracts in most cases. The predominant phenolic compounds in the extracts were oleuropein, hydrohytyrosol and rutin.

## 1. Introduction

Olive leaves contain numerous phenolic compounds which provide many benefits for human health; thus, several studies have employed extraction processes for their recovery. Olive leaves come from olive cultivation (about 25 kg per tree annually) and they are recovered in notable amounts during industrial production of olive oil (about 10% of the weight of processed olives). In particular, for Greece, olive cultivation is an important agricultural activity, since is the third biggest producer of olive oil in the world. Olive leaves are of great interest for their high valuable phenolic compound content that can be used in the pharmaceutical, food and cosmetic sectors [1].

Phenolic compounds in olive leaves are usually obtained by extraction with polar solvents [2] and are grouped as secoiridoids (oleuropein), flavonoids including flavones (apigenin and luteolin), flavonols (rutin and quercetin), flavanols (catechin) and simple phenols (tyrosol, hydroxytyrosol, vanillin, vanillic acid, gallic acid, caffeic acid and verbascoside) [1,3]. Their content varies in olive leaves depending on the harvesting time, origin and variety of olive [4,5]. Oleuropein, a less polar compound, is the major phenolic compound that, present in amounts from 1 to 14 g/100 g in dry olive leaves, representing 20–60% of the total phenolic compounds [2,6,7,8]. Hydroxytyrosol is a strong polar phenolic compound in olive leaves; it results from hydrolysis of oleuropein and occurs in about 0.2 g/100 g in dry olive leaves [2,9].

Phenolic compounds in olive leaves are known for their health benefits; according to research studies, olive leaf phenolic extracts have been proven to have important in vitro and in vivo properties, including antioxidant activity [3,10], anti-proliferative effects on leukaemic cells [11], cytotoxic activities against human breast cancer cells [12] and anti-HIV [13], anti-fungal [14] and antimicrobiological [3] activities. Oleuropein is considered the most active compound of all the phenolic compounds of olive leaves; it can inhibit in vitro platelet activation [15] and has protective and/or lenitive activity against UVB-induced erythema [16]. Recently, oleuropein has received extra attention due to its anti-viral activity against SARS-CoV-2 (COVID-19) [17,18]. Additionally, hydroxytyrosol has shown lipid-lowering effects [19]. Therefore, there is a growing interest in the recovery of these bioactive polyphenolic compounds from olive leaves by extraction processes.

Several techniques have been adopted for extraction of olive leaf phenolic compounds. Conventional extractions with organic solvents (e.g., ethanol and methanol) and water have mostly been used; however, they present disadvantages due to low extraction efficiency and a long duration of extraction [20]. In order to overcome these limitations, new assisted methods have been recently developed for extraction of bioactive compounds from plant materials, such as microwave-assisted (MAE), ultrasound-assisted (UAE), homogenization (HAE) and high hydrostatic pressure (HHPAE). In the MAE method, microwave energy heats the moisture inside the plant cell, generating pressure that causes the cell wall to rupture. Thus, leaching of the bioactive compounds from ruptured cells to the surrounding solvent is facilitated, improving the extraction yield [12]. The method offers simplified manipulation and has reduced solvent and energy demands, making it environmentally friendly [21]. The UAE method based on the principle of acoustic cavitation is capable of causing damage to the cell walls of plant material and thereby facilitating the bioactive compounds’ release. It stands out as a sustainable alternative method that requires a moderate investment and low energy costs [22]. The HAE method is usually employed in highspeed homogenization processes; the high shear rate promotes the rupture of the plant cell and the consequent bioactive compounds’ release into the solvent. Comparing HAE with other extraction methods, it offers benefits such as higher efficiency and less time and energy consumption, making it environmentally friendly [23]. The HHPAE method operating at pressures varying from 100 to 1000 MPa causes acceleration of cell wall disruption by rapid pressure changes within a short time, and enhances the solvent penetration of the cell wall and the bioactive compounds’ release [24]. It has been confirmed to be faster and more effective than other extraction methods which are widely applied to the food industries [25].

Nowadays, a new type of solvent named natural deep eutectic solvents (NADES) has been expanding in popularity as a promising alternative to traditional organic solvents [26]. NADES are based on mixtures of commonly used components: a hydrogen acceptor (e.g., nontoxic quaternary ammonium salts) and a naturally derived uncharged hydrogen-bond donor (e.g., sugars, alcohols, amines, carboxylic acids and vitamins). The resulting eutectic mixture melts at a lower temperature than both of its individual components. NADESs possess useful properties such as adjustable viscosity, non-flammability, low volatility and solubility in water [27,28], and have been of growing interest for both research and industrial purposes [29]. Additionally, in various studies it has been proven that they were harmless to various cell lines, even at high concentrations [30]. There is an increasing number of studies on bioactive plant compound extraction, including phenolic acids and flavonoids by applying NADES [31,32]. In previous studies, NADES have been used for phenolic compound extraction from olive pomace providing good results [33,34]. Additionally, a few studies have demonstrated the use of NADESs with the aforementioned novel assisted extraction methods (HAE, MAE, UAE and HHPAE) in the extraction of olive leaf phenolic compounds [32,35].

Based on the above, the scope of this study was to compare novel extraction methods for olive leaf phenolic compounds using NADES as solvents and to establish an environmentally friendly extraction method for olive leaf phenolic compounds. Four NADES systems composed of choline chloride combined with maltose (CMA), glycerol (CGL), citric (CCA) and lactic acid (CLA) were applied by combining assisted extraction methods such as microwave (MAE), ultrasound (UAE), homogenate (HAE) and high hydrostatic pressure (HHPAE). The goal was to evaluate the extracts regarding their phenolic content and profile as well as their antioxidant activity.

## 2. Results and Discussion

### 2.1. Preparation and Physicochemical Properties of NADES

The physical or physicochemical properties of used NADES (Table 1) affect the phenolic compound extraction; thus, basic properties such as density, viscosity, refractive index and surface tension have been measured in a previous study [33]. It must be noted that the viscosities and the surface tensions of all tested NADES were higher than those of ethanol:water 70% *v*/*v* and water. CMA was the most viscous solvent.

### 2.2. Effect of NADES Type on Olive Leaf Extraction by Different Assisted Extraction Methods

#### 2.2.1. Microwave-Assisted Extraction (MAE)

**Figure 1:**
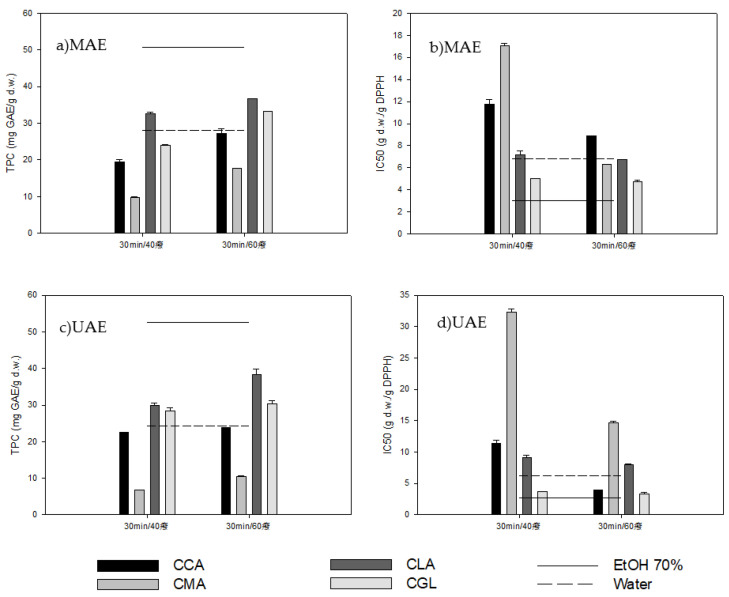
Effect of extraction temperature and NADES type on total phenolic content (TPC) (**a**,**c**) and antioxidant activity (IC50) (**b**,**d**) of olive leaf extracts by microwave-assisted extraction (MAE) and ultrasound-assisted extraction (UAE), respectively.

The effect of the NADES type and the extraction temperature on the results of MAE is shown in Figure 1a. The NADES type significantly affected the TPC of extracts (*p* < 0.05) with CLA showing the highest TPC (36.71 ± 0.07 mg GA/g d.w.). The findings are in contrast to those of Wei et al. (2015) [31], who reported that among various NADES examined, the choline choride/maltose mixture possessed excellent extractability for both polar and weak polar phenolic compounds using MAE. The differences between various NADES extracts could be explained by their physicochemical properties (surface tension and viscosity) and polarity; polar olive leaf phenolic compounds are more efficiently extracted by polar NADES (CCA and CLA) than by the lower polarity ones (CMA and CGL) [36]. Moreover, the extracts’ TPC increased significantly (*p* < 0.05) with the increase in temperature (Figure 1a). Higher temperatures can enhance the phenolic compounds’ solubility and increase their diffusion rate into the solvent; thus, the mass transfer rate increases [32,37,38]. Furthermore, by decreasing the surface tension and viscosity of NADES at higher temperatures, the phenolic compounds’ release is improved due to the enhancement of their mass transfer in the solvent [39]. Figure 1b presents the antioxidant activity (IC50) of olive leaf extracts obtained by MAE using different NADES and temperatures. The maximum antioxidant activity was obtained with CGL at 60 °C (4.76 ± 0.1 g d.w./g DPPH). The increase in temperature did not significantly change the extracts’ antioxidant activity. The comparison between the conventional solvents and the above NADES showed that the ethanol:water 70% *v*/*v* mixture was significantly more efficient, resulting in extracts with higher TPC values and antioxidant activity than NADES, while using water as a solvent resulted in almost equal antioxidant activity and TPC values to the NADES (Figure 1a,b).

#### 2.2.2. Ultrasound-Assisted Extraction (UAE)

Figure 1c shows the effect of the NADES type and the extraction temperature on the application of UAE, with CLA possessing the highest TPC for olive leaf extracts (38.32 ± 1.44 mg GA/g d.w). As in the MAE method, the most polar NADES showed higher phenolic compound extraction abilities than the less polar CMA. The extracts’ TPC significantly increased (*p* < 0.05) with the increase in temperature from 40 °C to 60 °C (Figure 1c), as expected. The IC50 of olive leaf extracts obtained by UAE using different NADES and temperatures is shown in Figure 1d. The maximum antioxidant activity was obtained with CGL at 60 °C (3.32 ± 0.39 g d.w./g DPPH). The ethanol:water 70% *v*/*v* mixture was proven to be a significantly more efficient solvent compared to NADES, showing a higher TPC value and antioxidant activity. As far as the use of water as a solvent is concerned, as in the MAE method, it resulted in almost equal antioxidant activity and TPC values as the NADES.

#### 2.2.3. Homogenate-Assisted Extraction (HAE)

**Figure 2:**
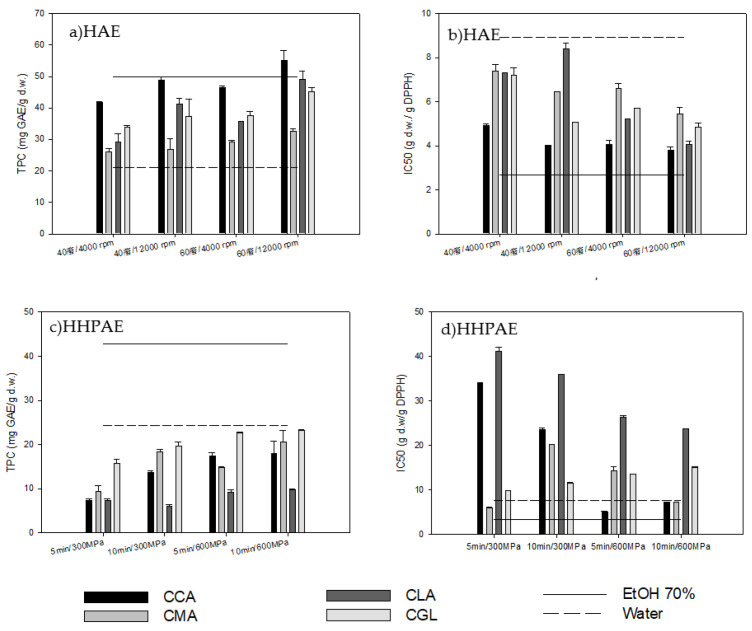
Effect of extraction temperature and NADES type on total phenolic content (TPC) (**a**,**c**) and antioxidant activity (IC50) (**b**,**d**) of olive leaf extracts by homogenate-assisted extraction (HAE) and high hydrostatic pressure-assisted extraction (HHPAE), respectively.

The effect of NADES, homogenization speed and extraction temperature on the application of HAE is shown in Figure 2a,b. The extracts’ TPC was significantly influenced by the NADES type, the homogenization speed and the temperature (*p* < 0.05). From the NADES examined, CCA again possessed the highest TPC (55.12 ± 1.08 mg GA/g d.w.). Moreover, when the temperature was increased from 40 °C to 60 °C, the olive leaf extracts’ TPC increased, as expected (Figure 2a). Additionally, higher homogenization speeds led to a higher mass transfer coefficient, thus facilitating the extraction process [40]. Furthermore, the IC50 was affected significantly (*p* < 0.05) both by the NADES type and the extraction temperature with the method using CCA at 12,000 rpm and 60 °C resulting in the extract with the maximum antioxidant activity (3.81 ± 0.15 g d.w./g DPPH) (Figure 2b). Finally, all NADES showed higher TPCs compared to the respective levels obtained with water. Moreover, the TPC of CCA and CLA extracts at 60 °C at 12,000 rpm were almost equal to those achieved by ethanol:water 70% *v*/*v*. Additionally, all NADES extracts showed better antioxidant activity compared to the water extract.

#### 2.2.4. High Hydrostatic Pressure-Assisted Extraction (HHPAE)

Figure 2c,d show the effect of NADES type, extraction time and pressure values after applying HHPAE on the antioxidant activity and the recovery of extracts’ phenolic compounds, respectively. The NADES type significantly influenced the extracts’ TPC (*p* < 0.05), with CGL treatment resulting in the highest TPC value (23.29 ± 0.08 mg GA/g d.w.). Additionally, the pressure increase led to increased TPC values of the extracts (*p* < 0.05), which is in agreement with other work [41]. Furthermore, the IC50 was affected significantly (*p* < 0.05) by the NADES type; the maximum antioxidant activity was obtained by using CCA at 600 MPa for 5 min (4.95 ± 0.20 g d.w./g DPPH) (Figure 2d). HHPAE as a nonthermal treatment does not facilitate phenolic compound extraction with viscous NADES. By using conventional solvents (water and ethanol:water 70% *v*/*v*), higher antioxidant activities and TPCs were obtained by HHPAE compared to when using NADES.

### 2.3. Phenolic Profiles of the NADES Extracts

The HPLC phenolic compound profile of olive leaf extracts using NADES, in combination with MAE, UAE, HAE and HHPAE are presented in Table 2a,b and Table 3a,b, respectively. It can be observed that the NADES type and the assisted extraction method significantly influenced the phenolic profile of the extracts (*p* < 0.05). In all extraction methods, the predominant phenolic compounds were oleuropein (OL), hydroxytyrosol (HT) and rutin (RU), while very small amounts of caffeic acid (CA), vanillin (VA) and luteolin (LU) were detected.

Using MAE, the highest level of OL was detected by using ethanol:water 70% (*v*/*v*), followed by CGL and water, while HT was the predominant phenolic compound with CCA, CMA and CLA. Similar findings were observed by Garcia et al. (2016) [42]. Ethanol and CGL extracted the maximum amount of RU. The sugar-based NADES (CMA) showed the lowest amounts of olives leaf phenolic compounds, due to its increased viscosity. The NADES type significantly influenced (*p* < 0.05) the total amount of phenolic compounds; CLA extracts possessed the highest amount of phenolic compounds.

In UAE, the NADES type significantly influenced the individual phenolic compounds extracted and their total amount (*p* < 0.05), similar to MAE (Table 2b). The maximum amount of OL and RU and the total amount of identified phenolic compounds were detected in CLA extract. The results are in accordance with previous studies, which reported that the NADES based on organic acids are most polar, which enhanced their ability to extract phenolic compounds [2,27,36]. The ethanol extract contained the highest amount of OL from all UAE extracts, but the amount of HT was limited. Furthermore, the water extract contained the lowest amounts of phenolic compounds, since only small amounts of OL and CA were detected.

In HAE, LU was not detected, except in the case using water as a solvent. HT was not detected in CGL and ethanol extracts (Table 3a). CCA proved to be the most efficient solvent at 12,000 rpm and 60 °C, showing the highest amount of OL and total amount of identified phenolic compounds, even more than when ethanol was used as a solvent. The increase in homogenization speed and temperature significantly enhanced the partial and total amounts of phenolic compounds (*p* < 0.05) but did not affect the ratio between OL and HT. The increase in homogenization speed led to higher mass transfer coefficient as mentioned before, thus enhancing the extraction of individual phenolic compounds. Additionally, at a higher temperature, the solvent viscosity decreased and the mass diffusivity increased, improving the release of olive leaf phenolic compounds.

Using HHPAE, the levels of phenolic compounds in the NADES extracts were similar to that of water, while ethanol proved to be the most efficient solvent for OL and total phenolic compounds (Table 3b). The predominant phenolic compounds were similar to all the above extraction methods; it should be underlined that LU was only detected using NADES, proving that they enhanced the diversity of obtained phenolic compounds. Additionally, the extractability of HT was increased using NADES. The increase in time and pressure in HHPAE significantly enhanced the total amount of phenolic compounds in the extracts (*p* < 0.05). Additionally, in this extraction method, the most polar organic acid-based NADES possessed enhanced extractability of phenolic compounds. Specifically, the highest amount of total amount of phenolic compounds was detected with CCA at 600 MPa for 10 min (13.6 ± 0.20 mg/g d.w.).

### 2.4. Statistical Analysis

**Figure 3:**
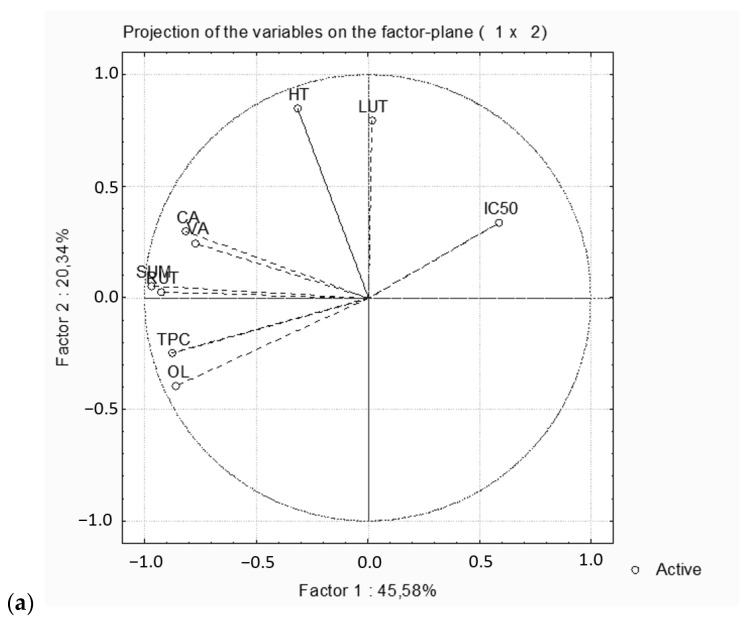

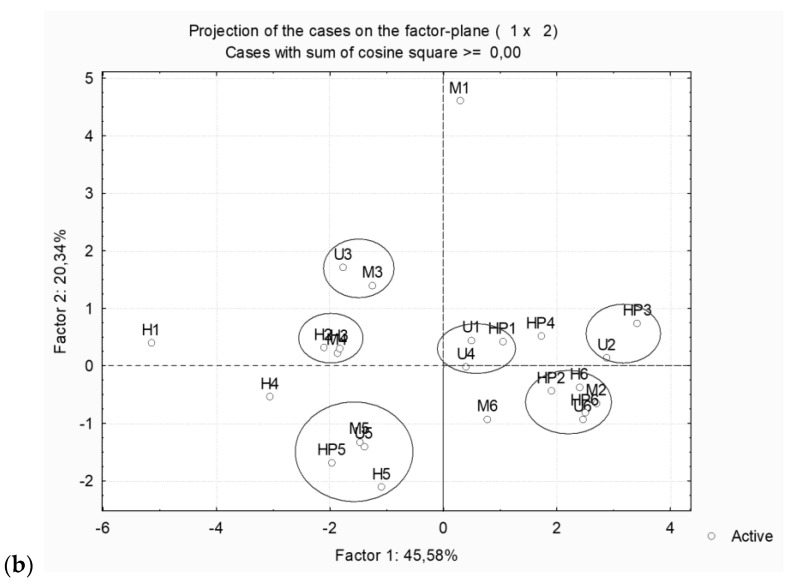
Principle component analysis of the dependent variables of the studied extractions (**a**) and of the samples with NADES (1 = CCA, 2 = CMA, 3 = CLA, 4 = GGL), ethanol:water 70% *v*/*v* (5) and water (6) as solvents, under intense conditions for each extraction method (M = MAE: 60 °C/30 min, U = UAE: 60 °C/30 min, H = HAE: 60 °C/12,000 rpm and HP = HHPAE: 600 MPa/10 min) (**b**).

Principal components analysis (PCA) was used to test the correlation of the TPC, the antioxidant activity, the individual extracted phenolic compounds (OL, HT, CA, VA, RU and LU) and the total amount of phenolic compounds (SUM) by using NADES in combination with the new assisted extraction methods including MAE, UAE, HAE and HHPAE (Figure 3a,b). Each point on the loading plot exhibits the contribution of a variable (TPC, antioxidant activity or individual phenolic compounds) to the score, while each point on the score plot exhibits a tested sample. The first principal component (PC1) describes 45.58% of the variation in extraction experiments, while the second principal component (PC2) describes 20.34%, contributing 65.92% of the extraction experiments’ total variation.

According to the PCA plot, the total amount of identified phenolic compounds and the TPC are strongly loaded in the first principal component, whereas the HT is positively correlated in the second principal component. According to the PCA score plot of the studied extractions, six main groups of samples were noted. The groups are (A) M3 and U3, (B) H2, H3 and M4, (C) HP5, M5, U5 and H5, (D) U1, HP1 and U4, (E) U2 and HP3 and (F) H6, HP6, U6, HP2 and M2.

It can be concluded that the group (A), samples using CLA as solvent and MAE and UAE as assisted extraction methods, exhibited the maximum amount of HT in the extracts, confirming that the organic acid NADES enhanced the extractability of simple phenols, which are the most polar phenolic compounds presenting in olive leaves [2]. Group (B), the extracts obtained with HAE using CMA and CLA as solvents were similar to that obtained with MAE using CGL in terms of antioxidant activity, TPC and the total amount of phenolic compounds. Additionally, group (C) consisted of the ethanol samples with the highest TPC, antioxidant activity, OL and total amount of phenolic compounds, indicating that the ethanol was the most efficient extracting medium. Group (D) had similar results concerning antioxidant activity and the total amount of phenolic compounds. The extracts in group (E) had the highest IC50 and the lowest antioxidant activity. Finally, group F consisted of the water extracts and had the lowest OL content and total amount of phenolic compounds, indicating that water was inefficient as a solvent for olive leaf phenolic compounds. In conclusion, the results described by using principal components analysis are in accordance with the results discussed above.

### 2.5. Comparison of Assisted Extraction Methods

Besides the use of alternative solvents, one of the criteria for an environmentally friendly extraction is to reduce energy consumption by using innovative technologies such as MAE, UAE, HAE and HHPAE [43]. Ultrasound, microwave and high hydrostatic pressure have been recognized as outstanding energy sources to promote extraction, increase extraction yield with high product quality, and to decrease extraction time [44]. The best extraction method for olive leaf phenolic compound recovery was proven to be HAE followed by MAE, while HHPAE gave the poorest results. A possible reason for this was that in HAE and MAE, the use of stirring enhances the phenolic compound extraction. The results are in accordance with previous studies [45]. HHPAE was proven to be an inappropriate method for use with NADES, probably due to limitations in temperature. However, the MAE, UAE and HAE could heat NADES in a short time, decreasing viscosity and surface tension, which were helpful for target compound extraction.

## 3. Materials and Methods

### 3.1. Raw Materials

Olive leaves (*Olea europaea* L. var.*argentata*) (initial moisture 49% *w*/*w*) were collected from the region of Thiva (Viotia, Greece). They were air dried at 35 °C for 24 h applying an airstream (final moisture 5% *w*/*w*), ground to 1 mm with a cutting mill (Pulverisette 15 cutting mill, FRITSCH, Idar-Oberstein, German) and kept at 4 °C until further use. The moisture (initial and final) of the olive leaves was measured gravimetrically using the method AOCS Ai-2-75 [46].

### 3.2. Chemicals and Reagents

Folin–Ciocalteu’s reagent, acetic acid, gallic acid, 2,2-diphenyl-1 picrylhydrazyl (DPPH), methanol (HPLC grade), ethanol, water (HPLC grade), acetonitrile (HPLC grade), sodium carbonate, sodium acetate and maltose (>97.0%) were purchased from Sigma Aldrich Chemical Co. (St. Louis, MO, USA). Phenolic standards: hydroxytyrosol, caffeic acid, vanillin, luteolin and rutin were supplied from Sigma-Aldrich (St. Louis, MO, USA) and oleuropein was purchased from Extrasynthese (Genay, France). Choline chloride (>98.0%) and lactic acid (>98.0%) were obtained from Acros Organics (Geel, Belgium), citric acid (>98.0%) was purchased from Univar (LaiwuTaihe Biochemistry Co. Ltd., Laiwu, China) and glycerol (>99.0%) was purchased from Lach-Ner (Neratovice, Czech Republic).

### 3.3. NADES Preparation

The following NADES–water mixtures were prepared according to Sofia Chanioti and Tzia (2018) [33]: choline chloride–citric acid (CCA), choline chloride–maltose (CMA), choline chloride–lactic (CLA) and choline chloride–glycerol (CGL) acid (Table 1) and referred to in the text as NADES.

### 3.4. Olive Leaf Phenolic Compound Extraction

An amount of dried ground olive leaves were mixed with a predetermined volume of NADES in an extraction vessel and the mixture was extracted with the selected assisted methods. The solid/liquid ratio was 1/12.5 g/mL. After extraction, the mixture was centrifuged at 10,000 rpm for 10 min and the supernatant (extract) was collected. Each experiment was performed in triplicate. The extracts were evaluated for their total phenolic content (TPC), antioxidant activity and individual phenolic compounds using HPLC. The conditions of the extraction experiments were selected according to preliminary studies.

#### 3.4.1. Microwave-Assisted Extraction (MAE)

Microwave-assisted extraction (MAE) was carried out at 200 W using laboratory equipment (Nanjing Xianou Instruments Manufacture Co., Ltd., Maixiang Science Park, Xixia Area, Nanjing, China) at certain temperatures (40 °C or 60 °C) for 30 min duration. The desired extraction temperature was controlled by regulating the nominal microwave power. Temperature and microwave radiation were constantly monitored during the process.

#### 3.4.2. Ultrasound-Assisted Extraction (UAE)

Ultrasound-assisted extraction (UAE) was performed in an ultrasound bath (Elmasonic S30 (H) 60 kHz, 280 W, Elma Schmidbauer GmbH, Singen (Hohentwiel), Germany) at certain temperatures (40 °C or 60 °C) for 30 min duration. A constant extraction temperature was achieved via circulating water.

#### 3.4.3. Homogenate-Assisted Extraction (HAE)

For homogenate-assisted extraction (HAE), a highspeed homogenizer (UnidriveX1000 Homogenizer Drive, CAT, Elma Schmidbauer GmbH, Germany) was used at certain temperatures (40 °C or 60 °C) and homogenization speeds (4000 or 12,000 rpm) for 30 min duration.

#### 3.4.4. High Hydrostatic Pressure-Assisted Extraction (HHPAE)

High pressure extraction was conducted using laboratory pilot scale HHP equipment (Food Pressure Unit FPU 1.01, Resato International, Roden, Holland) under certain conditions of pressure (300 and 600 MPa) and duration (5 and 10 min) at 25 °C.

Additionally, comparative experiments were carried out with the same solid/liquid ratio (1/12.5 g/mL) with conventional solvents, water and ethanol:water 70% *v*/*v* under the intense conditions of each extraction assisted method (MAE: 60 °C/30 min, UAE: 60 °C/30 min, HAE: 60 °C/12,000 rpm and HHPAE: 600 MPa/10 min).

### 3.5. Total Phenolic Content (TPC)

The determination of total phenolic content was achieved by the Folin–Ciocalteu method, as proposed by Waterhouse (2002) [47], using gallic acid as a standard. Data were expressed as mg gallic acid equivalents (GAE) per gram of dry olive leaves (mg GAE/g raw material (d.w.)) The NADES were examined in the Folin–Ciocalteu assay and negligible interference was found in the Folin–Ciocalteu assay, even for NADES which contained sugars.

### 3.6. Antioxidant Activity

Antioxidant activity was determined as proposed by Brand-Williams, Cuvelier and Berset (1995) [48], using the DPPH assay. Data were expressed as IC50 (g dry olive leaves (d.w.)/g DPPH). IC50 is the half maximal inhibitory concentration of extract that declines the initial concentration of DPPH by 50%. The NADES were examined in the DPPH assay and negligible interference was also found in the DPPH.

### 3.7. HPLC–DAD Analysis

HPLC-analysis was carried out on a HP 1100 Series gradient HPLC system (Agilent Technologies, Santa Clara, CA, USA) equipped with quaternary pump, diode array detector (Hewlett-Pachard, Waldbronn, Germany), an Agilent 1100 G1379A Vacuum Degasser, a CTO-10AS column oven (251 °C) and data analysis software (ChemStation for LC3D Software, Agilent 10 Technologies, Waldbronn, Germany). A column (250 × 4.6 mm) packed with 5 μm particle Hypersil C18 (MZ Analysentechnik, Mainz, Germany) was used. The analysis of phenolic compounds was performed as proposed by Japón-Luján (2006) [7]. Phenolic compounds were identified and quantified using reference curves of standards at the wavelength of maximum absorbance for each compound. The level of phenolic compounds was expressed in mg per g of dry olive leaves (d.w).

### 3.8. Statistical Analysis

The experimental results were evaluated by analysis of variance (ANOVA) using STATISTICA 7 (Statsoft Inc., Tulsa, OK, USA), while significant differences of expected values were estimated at the probability level *p* < 0.05. Principal component analysis (PCA) was also used to investigate the correlation between the independent and dependent variables of extraction experiments.

## 4. Conclusions

In this study, the combination of NADES with four assisted extraction methods was examined and compared. The NADES type significantly affected the total phenolic content of extracts; in MAE and UAE, choline chloride/lactic acid (CLA) solvent extraction resulted in the highest total phenolic content (TPC), while in HAE, choline chloride/citric acid (CCA) solvent extraction resulted in the highest total phenolic content (TPC) and in HHPAE, choline chloride/glycerol (CGL) solvent extraction resulted in the highest total phenolic content (TPC). Furthermore, in most cases, the ethanol extracts possessed higher total phenolic content than those of NADES. The predominant phenolic compounds were oleuropein (OL), hydrohytyrosol (HT) and rutin (RU), while a very small amount of caffeic acid (CA), vanillin (VA) and luteolin (LU) were detected. HT is a simple phenol, and was identified predominantly in the organic acid-based NADES extracts. The increase in temperature, speed and/or pressure promoted the extraction of olive leaf phenolic compounds. NADES are promising solvents, and have been proven to be efficient media for phenolic compound extraction from olive leaves; however, application and preparation of NADES should be accompanied by toxicological studies to allow for development of an environmentally friendly process.

The developed method based on the combination of different assisted extraction methods and NADES could be an alternative for phenolic compound extraction from olive leaves, as it provides higher extraction efficiency compared to conventional methods and achieves significantly reduced extraction times. The combination of the homogenate-assisted extraction method and NADES could be promising for the extraction of natural bioactive compounds.

## Figures and Tables

**Table 1:** Natural deep eutectic solvents (NADES) used in extraction experiments.

Code	Components	Mole Ratio	Water Addition (% *v*/*v*)
CCA	Choline chloride/Citric Acid	1:2	20
CLA	Choline chloride/Lactic Acid	1:2	20
CMA	Choline chloride/Maltose	1:2	20
CGL	Choline chloride/Glycerol	1:2	20

**Table 2:** Effect of NADES on the identified phenolic compounds (using HPLC) extracted from olive leaves using MAE (a) and UAE (b) and comparison with conventional solvents.

NADES	Extraction Conditions		Phenolic Compounds *						SUM ***
			Oleuropein (OL) **	Hydroxytyrosol (HT) **	Caffeic Acid (CA) **	Vanillin (VA) **	Rutin (RU) **	Luteolin (LU) **	
**(a) MAE**	**t (min)**	**T(°C)**							
**CCA**		40	3.32 ± 0.12 ^a^	6.41 ± 0.47	2.45 ± 0.34	0.59 ± 0.07	3.57 ± 0.38	0.05 ± 0.00	16.39 ± 0.71 ^a^
		60	n.d.	16.18 ± 0.35	0.06 ± 0.01	0.57 ± 0.05	4.06 ± 0.78	0.90 ± 0.05	21.77 ± 0.86 ^a^
**CMA**	30	40	n.d.	1.12 ± 0.10	0.01 ± 0.00	0.01 ± 0.00	0.04 ± 0.01	n.d.	1.18 ± 0.10 ^b^
		60	n.d.	1.12 ± 0.02	n.d.	n.d.	n.d.	n.d.	1.12 ± 0.02 ^b^
**CLA**	30	40	8.23 ± 1.19 ^b^	13.64 ± 0.07	0.07 ± 0.00	1.14 ± 0.10	5.89 ± 0.32	0.08 ± 0.01	29.05 ± 1.24 ^a^
		60	7.93 ± 1.07 ^b^	13.44 ± 0.39	0.06 ± 0.01	1.05 ± 0.07	5.73 ± 0.30	0.08 ± 0.02	28.29 ± 1.18 ^a^
**CGL**	30	40	11.27 ± 3.50 ^b^	1.56 ± 0.05	0.05 ± 0.01	0.26 ± 0.05	2.17 ± 0.10	0.18 ± 0.04	15.49 ± 3.50 ^a^
		60	14.61 ± 1.37 ^b^	2.64 ± 0.19	0.10 ± 0.02	1.58 ± 0.24	7.91 ± 0.59	0.14 ± 0.00	26.98 ± 1.52 ^a^
**EtOH 70%**	30	60	18.94 ± 0.50	n.d.	0.07 ± 0.01	n.d.	7.51 ± 0.03	0.08 ± 0.01	26.6 ± 0.50
**WATER**	30	60	10.60 ± 0.56	0.56 ± 0.10	0.02 ± 0.00	0.4 ± 0.03	3.77 ± 0.29	n.d.	15.35 ± 0.64
**(b) UAE**	**t (min)**	**T (°C)**							
**CCA**	30	40	4.46 ± 0.85 ^ab^	9.14 ± 0.12 ^bc^	0.04 ± 0.00	0.71 ± 0.05 ^ab^	2.5 ± 0.15 ^a^	0.06 ± 0.01	16.91 ± 0.87 ^b^
		60	5.02 ± 1.02 ^ab^	8.17 ± 0.20 ^bc^	0.04 ± 0.01	0.65 ± 0.00 ^ab^	3.11 ± 0.70 ^a^	0.06 ± 0.00	17.05 ± 1.25 ^b^
**CMA**	30	40	4.64 ± 1.23 ^a^	0.69 ± 0.05 ^a^	0.01 ± 0.00	0.15 ± 0.03 ^a^	0.65 ± 0.04 ^a^	0.01 ± 0.01	6.15 ± 1.23 ^a^
		60	2.83 ± 0.92 ^a^	2.96 ± 0.10 ^a^	0.01 ± 0.00	0.09 ± 0.03 ^a^	0.96 ± 0.03 ^a^	0.01 ± 0.01	6.86 ± 0.93 ^a^
**CLA**	30	40	8.80 ± 3.55 ^c^	9.58 ± 0.47 ^c^	0.04 ± 0.01	0.91 ± 0.03 ^b^	4.70 ± 0.22 ^b^	0.09 ± 0.01	24.12 ± 3.58 ^c^
		60	9.96 ± 3.21 ^c^	13.87 ± 0.50 ^c^	0.08 ± 0.01	1.38 ± 0.07 ^b^	7.02 ± 0.28 ^b^	0.13 ± 0.01	32.44 ± 3.26 ^c^
**CGL**	30	40	6.57 ± 0.55 ^bc^	2.96 ± 0.08 ^ab^	0.04 ± 0.00	0.67 ± 0.08 ^b^	0.49 ± 0.11 ^a^	0.12 ± 0.01	10.85 ± 0.57 ^ab^
		60	8.53 ± 0.63 ^bc^	5.07 ± 0.12 ^ab^	0.06 ± 0.01	0.81 ± 0.09 ^b^	0.64 ± 0.08 ^a^	0.07 ± 0.00	15.18 ± 0.65 ^ab^
**EtOH 70%**	30	60	19.89 ± 1.08	2.67 ± 0.00	0.03 ± 0.00	0.39 ± 0.00	5.91 ± 0.00	n.d.	28.89 ± 1.08
**WATER**	30	60	1.77 ± 0.05	n.d.	0.01 ± 0.00	n.d.	n.d.	n.d.	1.78 ± 0.05

* Mean value of three replicates ± standard deviation. ** mg/g d.w. *** the total amount of identified phenolic compounds. n.d.: not detected. Means within the same column followed by different letters (a, b, c) are significantly different (*p* < 0.05).

**Table 3:** Effect of NADES on the identified phenolic compounds (using HPLC) extracted from olive leaves using HAE (a) and HHPAE (b) and comparison with conventional solvents.

NADES	Extraction Conditions		Phenolic Compounds *						SUM ***
			Oleuropein (OL) **	Hydroxytyrosol (HT) **	Caffeic Acid (CA) **	Vanillin (VA) **	Rutin (RU) **	Luteolin (LU) **	
**(a) HAE**	**T (°C)**	**Speed (rpm)**							
**CCA**	40	4000	7.55 ± 0.10	12.63 ± 0.20 ^c^	0.08 ± 0.01	1.15 ± 0.08 ^a^	10.70 ± 0.35	n.d.	32.11 ± 0.42 ^b^
		12,000	7.61 ± 0.32	13.60 ± 0.10 ^c^	0.07 ± 0.01	1.29 ± 0.05 ^a^	11.08 ± 0.20	n.d.	33.65 ± 0.39 ^b^
	60	4000	19.07 ± 0.22	7.99 ± 0.18 ^c^	0.06 ± 0.02	0.53 ± 0.07 ^a^	5.01 ± 0.28	n.d.	32.66 ± 0.41 ^b^
		12,000	32.88 ± 0.08	10.07 ± 0.12 ^c^	0.20 ± 0.01	1.71 ± 0.06 ^a^	8.00 ± 0.75	n.d.	52.86 ± 0.77 ^b^
**CMA**	40	4000	13.22 ± 1.51	4.92 ± 1.33 ^b^	0.07 ± 0.00	1.49 ± 0.10 ^ab^	5.56 ± 0.36	n.d.	25.26 ± 2.05 ^a^
		12,000	14.24 ± 1.52	5.25 ± 0.33 ^b^	0.08 ± 0.01	1.18 ± 0.18 ^ab^	5.17 ± 0.01	n.d.	25.92 ± 1.57 ^a^
	60	4000	13.35 ± 1.01	5.26 ± 0.65 ^b^	0.09 ± 0.02	1.39 ± 0.11 ^ab^	5.57 ± 0.15	n.d.	25.66 ± 1.22 ^a^
		12,000	17.57 ± 0.19	6.40 ± 0.02 ^b^	0.10 ± 0.02	1.98 ± 0.27 ^ab^	5.91 ± 0.46	n.d.	31.96 ± 0.57 ^a^
**CLA**	40	4000	9.19 ± 1.30	3.24 ± 0.34 ^ab^	0.04 ± 0.01	0.92 ± 0.10 ^a^	5.76 ± 0.49	n.d.	19.15 ± 1.43 ^a^
		12,000	14.71 ± 0.88	n.d.	0.07 ± 0.02	0.92 ± 0.04 ^a^	8.41 ± 0.95	n.d.	24.11 ± 1.30 ^a^
	60	4000	14.52 ± 1.67	2.88 ± 0.00 ^ab^	0.10 ± 0.03	0.83 ± 0.33 ^a^	7.58 ± 1.48	n.d.	25.91 ± 2.26 ^a^
		12,000	12.47 ± 1.46	9.90 ± 0.93 ^ab^	0.08 ± 0.00	1.23 ± 0.04 ^a^	5.12 ± 0.60	n.d.	28.80 ± 1.83 ^a^
**CGL**	40	4000	14.64 ± 1.35	n.d.	0.08 ± 0.01	1.50 ± 0.06 ^b^	8.03 ± 0.46	n.d.	24.25 ± 1.43 ^a^
		12,000	15.65 ± 1.06	n.d.	0.09 ± 0.01	1.48 ± 0.05 ^b^	8.98 ± 1.06	n.d.	26.20 ± 1.50 ^a^
	60	4000	18.25 ± 0.46	n.d.	0.09 ± 0.03	2.16 ± 0.07 ^b^	8.19 ± 0.25	n.d.	28.69 ± 0.53 ^a^
		12,000	19.16 ± 1.15	n.d.	0.12 ± 0.02	2.34 ± 0.06 ^b^	8.55 ± 0.95	n.d.	30.17 ± 1.49 ^a^
**EtOH 70%**	60	12,000	26.67 ± 1.13	n.d.	n.d.	0.11 ± 0.02	4.97 ± 0.52	n.d.	31.75 ± 1.24
**WATER**	60	12,000	1.09 ± 0.06	1.23 ± 0.05	0.02 ± 0.00	0.29 ± 0.05	0.29 ± 0.03	0.02 ± 0.00	2.94 ± 0.10
**(b) HHPAE**	**HP** **(MPa)**	**t** **(min)**							
**CCA**	300	5	1.26 ± 0.26 ^c^	3.32 ± 0.08 ^c^	0.01 ± 0.00 ^ab^	0.24 ± 0.02	1.24 ± 0.02	0.02 ± 0.00 ^a^	6.09 ± 0.27 ^c^
		10	1.50 ± 0.19 ^c^	4.85 ± 0.10 ^c^	0.02 ± 0.00 ^ab^	0.35 ± 0.02	1.88 ± 0.02	0.03 ± 0.00 ^a^	8.63 ± 0.22 ^c^
	600	5	2.20 ± 0.27 ^c^	6.08 ± 0.06 ^c^	0.02 ± 0.00 ^ab^	0.41 ± 0.02	2.59 ± 0.05	0.03 ± 0.00 ^a^	11.33 ± 0.28 ^c^
		10	2.39 ± 0.09 ^c^	7.35 ± 0.06 ^c^	0.03 ± 0.00 ^ab^	0.61 ± 0.04	3.18 ± 0.16	0.04 ± 0.01 ^a^	13.6 ± 0.20 ^c^
**CMA**	300	5	0.35 ± 0.07 ^ab^	0.50 ± 0.02 ^a^	n.d.	0.70 ± 0.03	0.04 ± 0.00	0.01 ± 0.00 ^a^	1.60 ± 0.08 ^a^
		10	1.07 ± 0.35 ^ab^	0.33 ± 0.02 ^a^	0.01 ± 0.00 ^ab^	0.10 ± 0.03	0.85 ± 0.04	0.03 ± 0.00 ^a^	2.39 ± 0.35 ^a^
	600	5	1.03 ± 0.50 ^ab^	0.74 ± 0.02 ^a^	0.01 ± 0.00 ^ab^	0.21 ± 0.00	1.41 ± 0.03	0.04 ± 0.01 ^a^	3.44 ± 0.50 ^a^
		10	1.53 ± 0.05 ^ab^	0.69 ± 0.07 ^a^	0.03 ± 0.00 ^ab^	0.24 ± 0.06	1.92 ± 0.01	0.06 ± 0.01 ^a^	4.47 ± 0.11 ^a^
**CLA**	300	5	0.54 ± 0.02 ^a^	1.76 ± 0.01 ^b^	0.01 ± 0.00 ^a^	0.13 ± 0.00	0.76 ± 0.00	0.01 ± 0.00 ^a^	3.21 ± 0.02 ^ab^
		10	0.82 ± 0.08 ^a^	2.39 ± 0.00 ^b^	0.01 ± 0.00 ^a^	0.21 ± 0.01	0.82 ± 0.03	0.01 ± 0.00 ^a^	4.26 ± 0.09 ^ab^
	600	5	0.98 ± 0.10 ^a^	2.87 ± 0.00 ^b^	0.01 ± 0.00 ^a^	0.24 ± 0.01	1.30 ± 0.01	0.02 ± 0.00 ^a^	5.42 ± 0.10 ^ab^
		10	1.05 ± 0.04 ^a^	2.85 ± 0.01 ^b^	0.01 ± 0.00 ^a^	0.24 ± 0.01	1.22 ± 0.06	0.02 ± 0.00 ^a^	5.39 ± 0.12 ^ab^
**CGL**	300	5	0.52 ± 0.04 ^bc^	0.97 ± 0.01 ^b^	0.04 ± 0.01 ^b^	0.08 ± 0.02	0.15 ± 0.02	0.02 ± 0.00 ^b^	1.78 ± 0.05 ^b^
		10	2.60 ± 0.34 ^bc^	1.37 ± 0.03 ^b^	0.03 ± 0.00 ^b^	0.55 ± 0.01	2.43 ± 0.01	0.11 ± 0.01 ^b^	7.09 ± 0.34 ^b^
	600	5	1.06 ± 0.12 ^bc^	2.47 ± 0.13 ^b^	0.02 ± 0.01 ^b^	0.13 ± 0.01	0.19 ± 0.01	0.26 ± 0.02 ^b^	4.13 ± 0.18 ^b^
		10	2.22 ± 0.29 ^bc^	4.19 ± 0.01 ^b^	0.03 ± 0.00 ^b^	0.46 ± 0.02	1.66 ± 0.01	0.15 ± 0.02 ^b^	8.71 ± 0.29 ^b^
**EtOH 70%**	600	10	29.18 ± 0.87	n.d.	0.02 ± 0.00	1.03 ± 0.03	6.73 ± 0.55	n.d.	36.96 ± 1.03
**WATER**	600	10	1.31 ± 0.05	0.53 ± 0.02	n.d.	n.d.	0.67 ± 0,01	n.d.	2.51 ± 0.05

* Mean value of three replicates ± standard deviation. ** mg/g d.w. *** the total amount of identified phenolic compounds. n.d.: not detected. Means within the same column followed by different letters (a–c) are significantly different (*p* < 0.05).

## Data Availability

The data presented in this study are available on request from the corresponding author.

## Sample Availability

Samples of the compounds are not available from the authors.
